# Traumatic testicular dislocation: A reminder for the unwary

**DOI:** 10.4103/0974-2700.70762

**Published:** 2010

**Authors:** Pawan Vasudeva, Divakar Dalela, Dharamveer Singh, Apul Goel

**Affiliations:** Department of Urology, C.S.M. Medical University (Upgraded King George’s Medical College), Lucknow, Uttar Pradesh, India

Sir,

Traumatic testicular dislocation (TTD) is a rare complication of blunt abdominal trauma that is usually overlooked initially.[1] Delayed or missed diagnosis may be due to associated injuries or a lack of awareness of its possible occurrence. Delayed correction of TTD may result in infertility due to elevated temperature exposure leading to reduced spermatids, spermatogonia and relatively increased sertoli cells.[2]

A 17-year-old male motorcyclist presented with acute right groin pain after a low-velocity head-on-collision. He was hemodynamically stable, had a well-developed empty right hemiscrotum with an ovoid tender swelling in the ipsilateral groin and overlying skin ecchymosis [Figure 1a]. His left testis was normal and he reported a previous scrotal position of both testes. Color Doppler ultrasound confirmed the diagnosis of a dislocated right testis with its adequate blood supply. On immediate surgical exploration, the right testis was lying in the superficial inguinal pouch and was macroscopically normal [Figure 1b]. The scrotal hematoma was evacuated and right testis was reposed back to the scrotum without fixation. At 6-month follow up, both the testes were equal sized and in their normal intrascrotal position.

**Figure 1:**
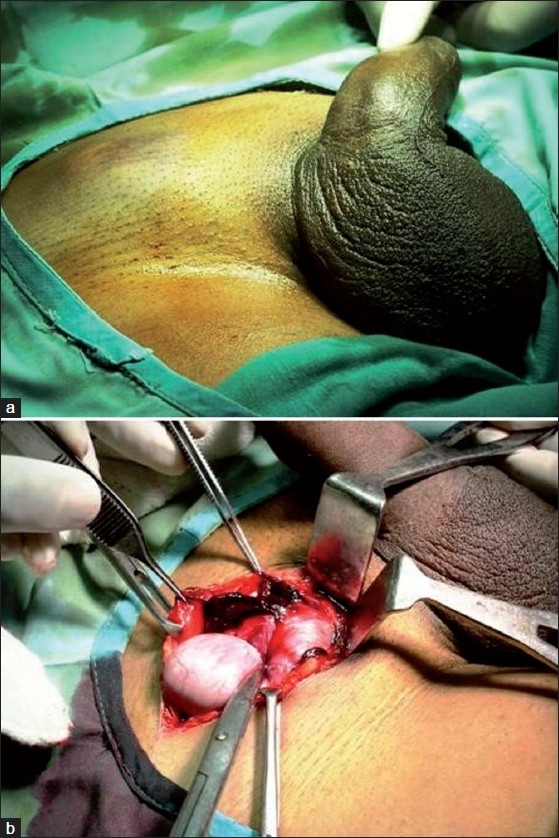
Visible swelling in the right groin with ecchymosis of the overlying skin (a), and the right testis appears to be normal (b)

Testicular dislocation is defined as the displacement of normally located testis to a position other than the scrotum. TTD is usually related to straddle injuries from motorcycle accidents where the rider is propelled forward, with the scrotum and perineum striking the fuel tank. The shape of fuel tank is such that it drives a smooth wedge into the groin area, forcibly displacing each testis in supero-lateral direction.[3] In trauma setting, predisposing factors to dislocation may include an associated indirect inguinal hernia or small-sized testis.

Dislocation may be unilateral or bilateral, superficial (testis is forced into superficial inguinal pouch) or internal (testis is forced through the external ring into the inguinal canal or even abdominal cavity). Possible sites with relative frequency are: superficial inguinal 50%, pubic 18%, penile 8%, canalicular 8%, truly abdominal 6%, perineal 4%, acetabular 4% and crural 2%.[4]

Finding of an empty hemiscrotum on palpation is the key toward establishing diagnosis, though a scrotal hematoma may mask it. Differential diagnosis includes an undescended testis, retractile testis, or traumatic torsion testis with high lying testis. Doppler ultrasound scan should be performed to confirm intact viable testis and to exclude coexisting problems, i.e. testicular rupture, torsion or hematoma.[5]

Closed reduction may be attempted as the initial treatment; however, associated testicular torsion or rupture is a contraindication for closed reduction and these should be ruled out by imaging before attempting closed reduction. Successful testicular reduction means testicle in its normal anatomic intrascrotal location and immediate pain relief. Surgical exploration is preferred because of possibility of testicular torsion/rupture, high failure rate of closed reduction and minimal surgical morbidity.[6] Surgical exploration also permits thorough evacuation of hematomas and avoids any chance of iatrogenic torsion testis while doing closed reduction, a potential danger which necessitates post-reduction Doppler to exclude it. We do not find any need for additional testicular fixation after reposing the testis, as the dissection done to evacuate the blood that has dispersed into tissues and consequent healing of contused tissue will eventually generate scrota-testicular adhesions.

This case emphasizes upon thorough evaluation of trauma patients in emergency room including routine bilateral testicular palpation during secondary survey to avoid delayed or missed diagnosis of testicular dislocation, especially in victims of motorcycle accidents.

## References

[1] Ko SF, Ng SH, Wan YL, Huang CC, Lee TY, Kung CT (2004). Testicular dislocation: an uncommon and easily overlooked complication of blunt abdominal trauma. Ann Emerg Me d.

[2] Hayami S, Ishigooka M, Suzuki Y, Sasagawa I, Nakada T, Mitote K (1996). Pathological changes of traumatic, dislocated testis. Urol Int.

[3] Pollen JJ, Funckes C (1982). Traumatic dislocation of the testes. J Trauma.

[4] Schwartz SL, Faerber GJ (1994). Dislocation of the testes, as a delayed presentation of scrotal trauma. Urology.

[5] Shefi S, Mor Y, Dotan ZA, Ramon J (1999). Traumatic testicular dislocation: a case report and review of published reports. Urology.

[6] Edson M, Meek JM (1979). Bilateral testicular dislocation with unilateral rupture. J Urol.

